# BRCA1 regulates insulin-like growth factor 1 receptor levels in ovarian cancer

**DOI:** 10.3892/ol.2014.1929

**Published:** 2014-02-28

**Authors:** BO LIU, DA LI, YI-FU GUAN

**Affiliations:** 1 Department of Biochemistry and Molecular Biology, China Medical University, Shenyang, Liaoning 110001, P.R. China; 2 Experimental Research Center, Shengjing Hospital of China Medical University, Shenyang, Liaoning 110004, P.R. China

**Keywords:** breast cancer 1, insulin-like growth factor 1 receptor, ovarian cancer

## Abstract

Breast cancer 1 (BRCA1) and insulin-like growth factor 1 receptor (IGF1R) are critical in ovarian cancer progression. However, the crosstalk between the BRCA1 and IGF1R signaling pathways in ovarian cancer remains largely unknown. The effects of BRCA1 on IGF1R were assessed in 121 serous ovarian cancer patients (BRCA1 mutation, n=30; non-BRCA1 mutation, n=32; hypermethylated BRCA1 promoter, n=28; and non-methylation, n=31). BRCA1 promoter methylation was analyzed via bisulfite sequencing using primers focused on the core promoter region. The expression levels of BRCA1 and IGF1R were assessed by immunohistochemistry and real-time polymerase chain reaction. Knockdown and overexpression of BRCA1 were achieved using a lentiviral vector in 293T and SKOV3 ovarian cancer cells, and primary non-mutated and BRCA1-mutated ovarian cancer cells. The present study demonstrated that IGF1R expression is increased in non-BRCA1-mutated ovarian cancer when compared with adjacent normal tissue. Furthermore, IGF1R levels are additionally significantly elevated in BRCA1 inactivation ovarian cancer (BRCA1 mutation or hypermethylated BRCA1 promoter). In addition, BRCA1 knockdown was found to be an effective method of activating IGF1R expression in non-BRCA1-mutated ovarian cancer cells. The observations of the current study indicate that BRCA1 may be a potential trigger that is involved in the transcriptional regulation of IGF1R in the development of ovarian cancer.

## Introduction

Ovarian cancer is characterized by a high mortality rate among the gynecological malignancies worldwide (1). To date, although the exact cause of ovarian cancer remains largely unknown, breast cancer (BRCA) mutations are the only known hereditary factors. The risk of ovarian cancer conferred by BRCA mutations has been found to be regulated by genetic and environmental components (2,3). Insulin-like growth factor 1 receptor (IGF1R) is a tyrosine kinase receptor that exerts a direct effect on cell proliferation, transformation, metastasis, invasion and resistance to anticancer therapies (4). Recently, IGF1R has drawn considerable interest in the field of epithelial neoplasms, in particular, several types of gynecological malignancies, including ovarian cancer (5). Notably, emerging evidence has established the following: i) IGF1R is a potential link between genetic and environmental interactions (3,6); ii) convergence exists between IGF1R-related cell proliferation pathways and BRCA1-related tumor protective pathways (3); and iii) BRCA1 mutation-related cancer may be regulated by the IGF1R signaling pathway (3). Therefore, understanding the complex interrelationship between IGF1R and BRCA1 may improve the current understanding of the basic molecular mechanism of ovarian cancer. As a result, the present study was undertaken to investigate IGF1R expression from genetic (BRCA1 mutation) and epigenetic (BRCA1 promoter methylation) aspects in ovarian cancer and to provide novel insights into the regulatory mechanism involving IGF1R.

## Patients and methods

### Patients and tissue collection

The present study was approved by the Institutional Review Board at the China Medical University (Shenyang, China). In total, 121 serous ovarian cancer patients (BRCA1 mutation, n=30; non-BRCA1 mutation, n=32; hypermethylated BRCA1 promoter, n=28; and non-methylation, n=31) were enrolled between 2010 and 2012, and all patients provided written informed consent. Fresh tumor samples, adjacent normal ovarian tissue, ascites and blood samples were obtained at the time of primary surgery prior to any chemotherapy or radiotherapy. Hematoxylin and eosin staining of the samples for histopathological diagnosis and grading were determined by three pathologists using the World Health Organization criteria (7). All patients were screened for BRCA1 mutations by multiplex polymerase chain reaction (PCR) with complete sequence analysis as previously reported (8).

### Cell culture and lentiviral infection

Primary ovarian cancer cells were obtained from ascites of patients undergoing surgery for ovarian cancer and were cultured in RPMI-1640 with 10% fetal bovine serum (FBS; Invitrogen Life Technologies, Carlsbad, CA, USA), as described previously (9). Human 293T and SKOV3 ovarian cancer cells were maintained in Dulbecco’s modified Eagle’s medium with 10% FBS (Invitrogen Life Technologies). Lentiviral vectors expressing short hairpin RNAs against BRCA1 (cat. no. NM_007299) were obtained from GeneChem Co., Ltd. (Shanghai, China) and synthesized as follows: Forward, 5′-CcggaaCCTGTCTCCACAAAGTGTGCTCGA GCACACTTTGTGGAGACAGGTTTTTTTg and reverse, 5′-aattcaaaaaaaCCTGTCTCCACAAAGTGTGCTCGAGCACACTTTGTGGAGACAGGTT. The non-silencing small interfering RNA sequence (TTCTCCGAACGTGTCACGT) served as a negative control. For overexpression of BRCA1, the open reading frame of BRCA1 (cat no. NM_007299) was cloned into the lentiviral vector GV287 (Ubi-MCS-3FLAG-SV40-EGFP; GeneChem Co., Ltd.). Transfections were performed using the polybrene and enhanced infection solution (GeneChem Co., Ltd.), according to the manufacturer’s instructions.

### Real-time PCR (qPCR) and immunohistochemistry analysis

qPCR and immunohistochemistry were performed as previously described (8). The specific primer sequences used for real-time PCR were as follows: Forward, 5′-TGATCCTGGATGCGGTGTCCAATA-3′ and reverse, 5′-TGGTCTTCTCACACATCGGCTTCT-3′ for IGF1R; forward, 5′-GGCTATCCTCTCAGAGTGACATTT-3′ and reverse, 5′-GCTTTATCAGGTTATGTTGCATGG-3′ for BRCA1; and forward, 5′-AGGTGAAGGTCGGAGTCA-3′ and reverse, 5′-GGTCATTGATGGCAACAA-3′ for GAPDH. The primary antibody used for immunohistochemistry was rabbit anti-IGF1R of human origin (1:200; Santa Cruz Biotechnology, Inc., Santa Cruz, CA, USA). Immunostaining was evaluated by two independent pathologists, blinded to the identity of the subject groups. Area quantification was performed using a light microscope (Nikon Eclipse 80i, Nikon Corporation, Tokyo, Japan; magnification, ×400) and analyzed by Image-Pro Plus 6.0 (Media Cybernetics, Inc., Rockville, MD, USA). The intensity of the staining was divided into 10 units.

### Bisulfite sequencing

Genomic DNA extracted from ovarian cancer and normal ovarian tissue using a TIANamp Genomic DNA kit (Tiangen Biotech Co., Ltd., Beijing, China) was subjected to bisulfite conversion using the EZ DNA Methylation-Direct kit (Zymo Research Corporation, Irvine, CA, USA) according to the manufacturer’s instructions; the conversion efficiency was estimated to be ≥99.6%. The DNA was subsequently amplified by nested PCR and following gel purification, cloning and transformation into JM109 competent *Escherichia coli* cells (Takara Bio, Inc., Shiga, Japan), 10 positive clones of each sample were sequenced to ascertain the methylation patterns of each CpG locus. The following primers were used: Forward, 5′-TTGTAGTTTTTTTAAAGAGT-3′ and reverse, 5′-TACTACCTTTACCCAAAACAAAA-3′ for round I; and forward, 5′-GTAGTTTTTTTAAAGAGTTGTA-3′ and reverse, 5′-ACCTTTACCCAAAACAAAAA-3′ for round II. The conditions used were as follows: 95°C for 2 min; 40 cycles of 30 sec at 95°C, 30 sec at 56°C and 45 sec at 72°C; and 72°C for 7 min.

### Statistical analysis

Data are presented as means ± standard deviation. Statistical differences in the data were evaluated by Student’s t-test or one-way analysis of variance as appropriate. P<0.05 was considered to indicate a statistically significant difference.

## Results

### Differences in the expression patterns of IGF1R in non-mutated and BRCA1-mutated ovarian cancer

**Figure 1:**
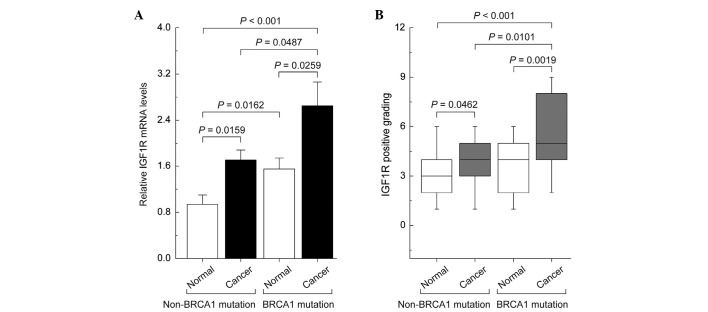
IGF1R expression patterns in non-mutated and BRCA1-mutated ovarian cancer. (A) Relative IGF1R mRNA levels were measured in non-mutated and BRCA1-mutated ovarian cancer, and their adjacent normal tissue. (B) IGF1R protein levels were assessed by immunohistochemistry in non-mutated and BRCA1-mutated ovarian cancer, and their adjacent normal tissue. Bar graphs are presented as the mean ± standard deviation. The intensity of the staining was divided into 10 units. IGF1R, insulin-like growth factor 1 receptor; BRCA1, breast cancer 1.

qPCR and immunohistochemical analysis showed that the levels of IGF1R mRNA and protein were increased in non-mutated and BRCA1-mutated ovarian cancer tissue compared with their adjacent normal tissue. However, BRCA1-mutated ovarian cancer markedly increased the expression of IGF1R compared with the remaining three groups (Fig. 1).

### Decreased expression of BRCA1 mediated by BRCA1 promoter hypermethylation is inversely correlated with IGF1R levels

**Figure 2:**
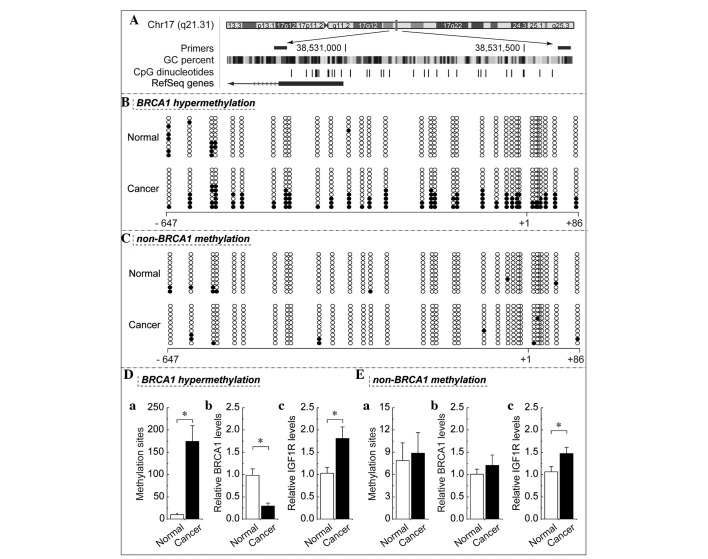
IGF1R expression patterns in ovarian cancer with hypermethylated promoter-mediated BRCA1 inactivation. (A) Location of CpG sites in the core promoter region of IGF1R. Genomic coordinates are shown, along with the primer amplified fragments, GC percentage, location of individual CpG dinucleotides (dashes) and IGF1R RefSeq gene (exon 1 shown as a black box and intron shown as an arrowed line). The arrow indicates the direction of transcription. (B and C) Comparative analysis of methylation patterns in the core promoter region of BRCA1 and their adjacent normal tissue. The circles correspond to the CpG sites denoted by the black dashes in Fig. 2A. Closed circles indicate methylated BRCA1 and open circles indicate unmethylated BRCA1. In total, 10 individual clones were sequenced for each sample. (Da and Ea) Summary of the methylation levels of BRCA1 core promoter from the measurements shown in B and C. (Db and Eb) Relative BRCA1 mRNA levels were measured in ovarian cancer with the identified hypermethylated or unmethylated BRCA1 promoter and compared with their adjacent normal tissue. (Dc and Ec) Relative IGF1R mRNA levels were measured in ovarian cancer with or without identified BRCA1 inactivation. Bar graphs are presented as the mean ± standard deviation. ^*^P<0.05 vs. normal. IGF1R, insulin-like growth factor 1 receptor; GC, guanine-cytosine; BRCA1, breast cancer 1.

**Figure 3:**
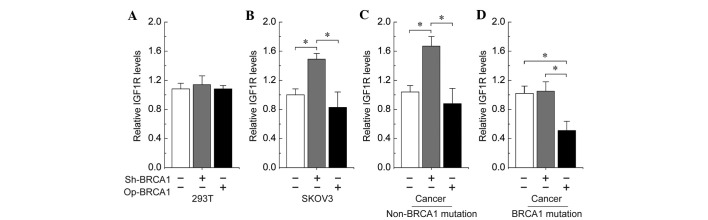
Effects of BRCA1 on IGF1R expression. (A–D) Relative IGF1R mRNA levels following overexpression or knockdown of BRCA1 in 293T, human SKOV3 ovarian cancer, and primary non-mutated and BRCA1-mutated ovarian cancer cells. Bar graphs are presented as the mean ± standard deviation. Sh, short hairpin RNAs; Op, overexpression; IGF1R, insulin-like growth factor 1 receptor; BRCA1, breast cancer 1.

In mammals, promoter methylation is an epigenetic modification involved in regulating gene expression (10). Consistent with this theory, the present study showed that ovarian cancer tissue with hypermethylated BRCA1 promoter (Fig. 2B and Da) exhibited decreased expression of BRCA1 (Fig. 2D) when compared with adjacent normal tissue. However, no significant differences in BRCA1 expression (Fig. 2Eb) were observed in ovarian cancer with unmethylated BRCA1 promoter (Fig. 2C and Ea) as compared with their adjacent normal tissue. Based on these considerations, the low levels of BRCA1 appeared to be mediated by promoter hypermethylation, establishing this an appropriate model to investigate the physiological correlation between BRCA1 and IGF1R. Notably, the expression levels of IGF1R were markedly increased alongside hypermethylated promoter-mediated BRCA1 deficiency in ovarian cancer (Fig. 2Dc). In addition, IGF1R expression was also increased in ovarian cancer tissue (Fig. 2Ec), with no significant difference identified between BRCA1 promoter methylation and expression (Fig. 2Ea and b); however, the increase was not significant when compared with that observed in ovarian cancer with BRCA1 deficiency. BRCA1 regulates IGF1R expression in ovarian cancer cells. To further confirm the role of BRCA1 in the regulation of IGF1R, the effects of overexpression or knockdown of BRCA1 were observed in 293T cells, the SKOV3 human ovarian cancer cell line, and primary ovarian cancer cells with and without identified BRCA1 mutations. The results indicated that no significant changes were identified in the expression of IGF1R following overexpression or knockdown of BRCA1 in 293T cells (Fig. 3A). Notably, the knockdown of BRCA1 was observed to be an effective method of inducing an increase of IGF1R levels in SKOV3 and non-BRCA1-mutated ovarian cancer cells (Fig. 3B and C). In addition, the overexpression of BRCA1 effectively decreased the expression of IGF1R in BRCA1-mutated ovarian cancer cells (Fig. 3D).

## Discussion

The present study reports the following associations between BRCA1 and IGF1R status in ovarian cancer cells: i) IGF1R expression is increased in non-BRCA1-mutated ovarian cancer; ii) BRCA1 inactivation (BRCA1 mutation and promoter hypermethylation) markedly increases the expression of IGF1R; and iii) BRCA1 knockdown is an effective method to activate the IGF1R gene. These results indicated that BRCA1 may be a potential regulator of IGF1R in ovarian cancer, although, a comparable phenomenon has been observed in breast cancer (11,12). Notably, the activation effect of BRCA1 was primarily observed in cells originating from ovarian cancer, however, 293T cells were insensitive to the overexpression or knockdown of BRCA1. Therefore, the induced expression of IGF1R may be the result of a complex interaction of specific factors in ovarian cancer cells. Notably, numerous previous studies have hypothesized that BRCA1 haploinsufficiency is more likely to result in cancer, due to an extraordinary ability for clonal growth and proliferation (4). IGF1R is also key in regulating cell growth and proliferation during cancer development (13). Therefore, it may be predicted that BRCA1 inactivation-related high levels of IGF1R may be involved in promoting ovarian cancer progression. An increasing quantity of data indicates that there is extensive crosstalk between BRCA1-associated signaling pathways and hormone receptor signaling pathways. For example, BRCA1 degrades the progesterone receptor (PR) by counteracting the action of progesterone (14) and inhibits PR activity by preventing the PR from binding to the progesterone response elements (15). By contrast, progesterone has previously been reported to induce the downregulation of BRCA1 (16). In addition, BRCA1 represses estrogen receptor (ER) activity by regulating the phosphoinositide 3-kinase/Akt pathway and the acetylation versus ubiquitination of ER (17,18). Conversely, zinc finger protein 423 and cathepsin O are involved in estrogen-dependent BRCA1 expression (19); Nelson *et al* (20) observed that IGF1R promotes the expression and regulates the stability of BRCA1, while the present study endorsed the theory that the IGF1R gene is a physiologically relevant downstream target for BRCA1.

In conclusion, the present study emphasizes the convergence of the IGF1R-mediated cell proliferation pathway and a BRCA1-mediated antitumor mechanism. However, further clarification of the complex interactions between the BRCA1 and IGF1R signaling pathways, at the transcriptional, post-transcriptional and epigenetic levels, may improve the current understanding of the basic molecular mechanism of ovarian cancer.

## References

[1] Lech A, Daneva T, Pashova S (2013). Ovarian cancer as a genetic disease. Front Biosci (Landmark Ed).

[2] Pruthi S, Gostout BS, Lindor NM (2010). Identification and management of women with BRCA mutations or hereditary predisposition for breast and ovarian cancer. Mayo Clin Proc.

[3] Werner H, Bruchim I (2012). IGF-1 and BRCA1 signalling pathways in familial cancer. Lancet Oncol.

[4] Samani AA, Yakar S, LeRoith D, Brodt P (2007). The role of the IGF system in cancer growth and metastasis: overview and recent insights. Endocr Rev.

[5] Bruchim I, Werner H (2013). Targeting IGF-1 signaling pathways in gynecologic malignancies. Expert Opin Ther Targets.

[6] Pfäffle R, Kiess W, Klammt J (2012). Downstream insulin-like growth factor. Endocr Dev.

[7] Scully R (1999). Histological Typing of Ovarian Tumours.

[8] Bi FF, Li D, Yang Q (2013). Promoter hypomethylation, especially around the E26 transformation-specific motif, and increased expression of poly (ADP-ribose) polymerase 1 in BRCA-mutated serous ovarian cancer. BMC Cancer.

[9] Szlosarek PW, Grimshaw MJ, Kulbe H, Wilson JL, Wilbanks GD, Burke F, Balkwill FR (2006). Expression and regulation of tumor necrosis factor alpha in normal and malignant ovarian epithelium. Mol Cancer Ther.

[10] Varley KE, Gertz J, Bowling KM (2013). Dynamic DNA methylation across diverse human cell lines and tissues. Genome Res.

[11] Hudelist G, Wagner T, Rosner M (2007). Intratumoral IGF-I protein expression is selectively upregulated in breast cancer patients with BRCA1/2 mutations. Endocr Relat Cancer.

[12] Maor S, Yosepovich A, Papa MZ, Yarden RI, Mayer D, Friedman E, Werner H (2007). Elevated insulin-like growth factor-I receptor (IGF-IR) levels in primary breast tumors associated with BRCA1 mutations. Cancer Lett.

[13] Chen X, Meng Q, Zhao Y (2013). Angiotensin II type 1 receptor antagonists inhibit cell proliferation and angiogenesis in breast cancer. Cancer Lett.

[14] Calvo V, Beato M (2011). BRCA1 counteracts progesterone action by ubiquitination leading to progesterone receptor degradation and epigenetic silencing of target promoters. Cancer Res.

[15] Katiyar P, Ma Y, Riegel A, Fan S, Rosen EM (2009). Mechanism of BRCA1-mediated inhibition of progesterone receptor transcriptional activity. Mol Endocrinol.

[16] Ansquer Y, Legrand A, Bringuier AF, Vadrot N, Lardeux B, Mandelbrot L, Feldmann G (2005). Progesterone induces BRCA1 mRNA decrease, cell cycle alterations and apoptosis in the MCF7 breast cancer cell line. Anticancer Res.

[17] Ma Y, Fan S, Hu C (2010). BRCA1 regulates acetylation and ubiquitination of estrogen receptor-alpha. Mol Endocrinol.

[18] Ma Y, Hu C, Riegel AT, Fan S, Rosen EM (2007). Growth factor signaling pathways modulate BRCA1 repression of estrogen receptor-alpha activity. Mol Endocrinol.

[19] Ingle JN, Liu M, Wickerham DL (2013). Selective estrogen receptor modulators and pharmacogenomic variation in ZNF423 regulation of BRCA1 expression: individualized breast cancer prevention. Cancer Discov.

[20] Nelson AC, Lyons TR, Young CD, Hansen KC, Anderson SM, Holt JT (2010). AKT regulates BRCA1 stability in response to hormone signaling. Mol Cell Endocrinol.

